# Identification of Core Genes and Screening of Potential Targets in Glioblastoma Multiforme by Integrated Bioinformatic Analysis

**DOI:** 10.3389/fonc.2020.615976

**Published:** 2021-02-24

**Authors:** Ji’an Yang, Qian Yang

**Affiliations:** ^1^ Department of Neurosurgery, Renmin Hospital of Wuhan University, Wuhan, China; ^2^ Division of Nephrology, Renmin Hospital of Wuhan University, Wuhan, China

**Keywords:** glioma, differential expression analysis, protein interaction network, classification, potential target

## Abstract

Glioblastoma multiforme is the most common primary intracranial malignancy, but its etiology and pathogenesis are still unclear. With the deepening of human genome research, the research of glioma subtype screening based on core molecules has become more in-depth. In the present study, we screened out differentially expressed genes (DEGs) through reanalyzing the glioblastoma multiforme (GBM) datasets GSE90598 from the Gene Expression Omnibus (GEO), the GBM dataset TCGA-GBM and the low-grade glioma (LGG) dataset TCGA-LGG from the Cancer Genome Atlas (TCGA). A total of 150 intersecting DEGs were found, of which 48 were upregulated and 102 were downregulated. These DEGs from GSE90598 dataset were enriched using the overrepresentation method, and multiple enriched gene ontology (GO) function terms were significantly correlated with neural cell signal transduction. DEGs between GBM and LGG were analyzed by gene set enrichment analysis (GSEA), and the significantly enriched Kyoto Encyclopedia of Genes and Genomes (KEGG) pathways involved in synapse signaling and oxytocin signaling pathways. Then, a protein-protein interaction (PPI) network was constructed to assess the interaction of proteins encoded by the DEGs. MCODE identified 2 modules from the PPI network. The 11 genes with the highest degrees in module 1 were designated as core molecules, namely, GABRD, KCNC1, KCNA1, SYT1, CACNG3, OPALIN, CD163, HPCAL4, ANK3, KIF5A, and MS4A6A, which were mainly enriched in ionic signaling-related pathways. Survival analysis of the GSE83300 dataset verified the significant relationship between expression levels of the 11 core genes and survival. Finally, the core molecules of GBM and the DrugBank database were assessed by a hypergeometric test to identify 10 drugs included tetrachlorodecaoxide related to cancer and neuropsychiatric diseases. Further studies are required to explore these core genes for their potentiality in diagnosis, prognosis, and targeted therapy and explain the relationship among ionic signaling-related pathways, neuropsychiatric diseases and neurological tumors.

## Introduction

Neuroepithelial tumors are collectively referred to as gliomas, which represent approximately 30% of all primary brain and other central nervous system (CNS) tumors and are the most common intracranial malignancies. Malignant gliomas grow rapidly and spread to brain tissue, leading to distinct surrounding brain edema and obvious focal symptoms. The annual incidence rate is more than three case per 100,000 people. Based on histological criteria, gliomas are generally graded by cell activity and aggressiveness on a scale of I to IV. They are divided into low-grade gliomas (WHO I-II; LGGs) and high-grade gliomas (WHO grade III-IV; HGGs). LGGs are well-differentiated gliomas. Although these tumors are not biologically benign, their prognosis is relatively good. HGGs, including glioblastoma multiforme (GBM), are poorly differentiated gliomas. Such tumors are malignant tumors with a poor prognosis. GBM is the most commonly occurring type of glioma, with a 5-year survival rate of approximately 5.6% (1). However, this classification system has high interobserver variability, and the survival rate varies greatly between patients with different disease grades (2). Advances in the molecular characterization of gliomas have rebuilt our understanding of their biological process and led to the development of new glioma classification systems (3). The approach of searching efficient biomarkers to improve the characteristics of clinically relevant subgroups may help address this dilemma.

In the past two decades, there have been many studies on establishing specific molecular classifications for gliomas. For example, low-grade gliomas without isocitrate dehydrogenase (IDH) mutations exhibit similar molecular and clinical characteristics to glioblastomas (4). However, patients with IDH mutant gliomas have a better prognosis, and gliomas with low-grade histology tend to develop slowly (5). The methylation status of the O(6)-methylguanine-DNA methyltransferase (MGMT) gene is not only helpful in determining the grade and prognosis of glioma but also an important predictive biomarker of the benefit from alkylating agent therapy in glioblastoma (6, 7). With the development of high-throughput technologies, such as microarrays, next-generation sequencing and single-cell sequencing, combined with the use of multidimensional analysis, the understanding of the biological processes of glioma has become increasingly deeper. An increasing number of microarray and sequencing data are being deposited in public databases such as the Gene Expression Omnibus (GEO, http://www.ncbi.nlm.nih.gov/geo/) and the Cancer Genome Atlas (TCGA, https://cancergenome.nih.gov/), which is convenient for data downloading and reanalysis. These accumulated massive datasets have facilitated the development of classifications of gliomas, especially glioblastomas, to facilitate diagnosis, prognosis prediction, and treatment management.

In this study, we downloaded data for a large number of glioma samples deposited in the GEO and TCGA databases and then analyzed the differentially expressed genes (DEGs) between gliomas (comparing the different grades of gliomas) and normal controls with a bioinformatics approach. Additionally, other methods, including gene ontology (GO)/Kyoto Encyclopedia of Genes and Genomes (KEGG) and gene set enrichment analysis (GSEA) pathway analyses, construction of a protein-protein interaction (PPI) network and identification of putative molecules, were used to analyze these data and to determine key genes and pathways. The specific flow of data preparing, processing and analysis is shown in **Supplemental Figure 1**. The aim of this study was to identify hub genes and core pathways and to provide potential candidate biomarkers for the diagnosis, survival prediction and targeted treatment of gliomas.

## Materials and Methods

### Glioma Data Sets

Two sets of GBM microarray data, GSE90598 and GSE83300, were downloaded from GEO database. The data preprocessing method was as follows: the downloaded data were in the format of log2-transformed quantile-normalized signal intensity. First, the probe was mapped to the gene, and the empty probe was removed. The maximum value was taken as the expression level of the gene when multiple probes corresponded to the same gene. We downloaded the TCGA-GBM and TCGA-LGG datasets from TCGA. The data types downloaded included read counts and fragments per kilobase of transcript per million mapped reads upper quartile (FPKM-UQ). The baseline characteristics of patients with LGG (n=511) and patients with GBM (n=154) from TCGA datasets are summarized in **Supplemental Table 1**. There is no significant difference between the two groups in gender. Compared with the LGG group, the GBM group had a lower proportion of patients older than 60 years old, and a lower IDH mutation and 1p/19q codeletion rate.

### Differentially Expressed Gene Screening

Differential analysis was performed on the GSE90598 dataset using the “limma” (8) package in R. The differential genes were filtered with Benjamini’s corrected P value <1e-^10-2^ and a fold change of >2-fold as the thresholds. The “DESeq2” (9) package in R was used to analyze and filter the differentially expressed genes between the TCGA-GBM and TCGA-LGG datasets by selecting Benjamini’s corrected P value <1e-^10-2^ and fold change more than 2 as thresholds. Subsequently, the intersection set was obtained and used as the final set of differential genes. The results were displayed *via* the “VennDiagram” (https://CRAN.R-project.org/package=VennDiagram) package of R. Based on the expression of DEGs in the GSE90598 dataset, cluster analysis was performed. Sample types included healthy human brain tissue samples, GBM patient tissue samples and astrocyte cell line samples.

### Gene Ontology and Pathway Enrichment Analysis of Differentially Expressed Genes

To describe the biological characteristics and functional annotations of DEGs, the “overrepresentation” method of the “clusterProfiler” (10) package in R was used to assess GO function and KEGG pathway enrichment, respectively. The top 10 resulting pathways sorted by p value are displayed. The “GSEA” method was used to identify the enriched KEGG pathways based on the expression profiles between the LGG and GBM subtypes.

### Protein–Protein Interaction Network Construction and Module Analysis

A protein interaction analysis of the DEGs was performed to obtain the PPI network in the Search Tool for the Retrieval of Interacting Genes/Proteins (STRING) database *via* the following website: https://string-db.org/ (11). The genes with the highest degrees were designated as the core genes. The PPI network results contained a total of 153 links. The resulting file was imported into Cytoscape (12) for editing and mapping. Two modules were obtained through the MCODE plug-in in Cytoscape. The genes contained in the main module (module 1) were assessed by KEGG pathway enrichment analysis to obtain the main functional pathways of the module.

### Survival Analysis of Putative Hub Genes

Through differential expression analysis and PPI network analysis, 11 core genes of pleomorphic glioblastoma were finally determined. Using the “survival” (https://CRAN.R-project.org/package=survival) package in R, the patients were divided into high and low expression groups by the quartiles of the expression of each core gene. Survival analysis was performed to assess the association of gene expression levels with survival time. In addition, to combine the expression values of multiple genes for survival prediction, the method described below was used: the expression value of each gene in each sample was standardized (Xij, where i represents the gene and j represents the sample). First, a z-score matrix was created by subtracting the mean between samples and dividing by the standard deviation to compare between genes:

$$z_{ij}=\frac{x_{ij}-{\bar{x}}_{i\cdot }}{\text{SD}(x_{i\cdot })}$$

Then, in each sample, the average value of the upregulated gene z-score in the core genes was subtracted from the average value of the downregulated gene z-score to obtain the final expression level characteristic index sj:

$$s_{j}=\sum_{i\in U}\frac{z_{ij}}{n_{U}}-\sum_{i\in D}\frac{z_{ij}}{n_{D}}$$

Finally, patients were divided into a high expression group, a low expression group and an intermediate expression group according to the core gene expression characteristic index (sj) of each sample. The R package “survival” was used to analyze patient survival.

Verification was performed with the GSE83300 dataset. Based on the expression levels of the 11 core genes, the patients were divided into a high expression group and a low expression group, and survival analysis was performed to generate survival curves.

### Drug Screening for Glioma Treatment

Based on the expression data of the core genes of GBM, combined with the information of all drug molecular targets in the DrugBank (13) database, the significance analysis was performed using hypergeometric tests to find the drugs that could have a therapeutic effect on gliomas. Through differential expression analysis and PPI network analysis, k core genes were selected among the total N genes. Among them, n genes were related to a drug, and M genes had a probability distribution that obeyed the hypergeometric distribution. According to the significance of the P value, the relevance of the selected core genes to the drug was judged. The formula is as follows:

$$p(k)=P(X=k)=\frac{\left(\underset{k}{M}\right)*\left(\underset{n-k}{N-M}\right)}{\left(\underset{n}{N}\right)}$$

## Results

### Data Preprocessing and Differential Gene Identification

Two GBM microarray datasets (GSE83300 and GSE90598) were downloaded from the GEO database. The GSE90598 dataset contained 16 GBM and 9 normal samples, and the GSE83300 dataset included 50 GBM patient samples, as shown in **Table 1**. The downloaded data were preprocessed to log2-transformed quantile-normalized signal intensity format. The TCGA-GBM and TCGA-LGG datasets were obtained from the TCGA database, as shown in **Table 2**. The data types downloaded included read counts and FPKM-UQ.

**Table 1:** Sample information from GEO database.

Dataset ID	Platform	GBM	Normal
GSE90598	GPL17692	16	9
GSE83300	GPL6480	50	0

**Table 2:** GBM and LGG expression profile datasets downloaded from TCGA database.

Dataset ID	GBM	LGG
TCGA-GBM	154	0
TCGA-LGG	0	511

**Figure 1:**
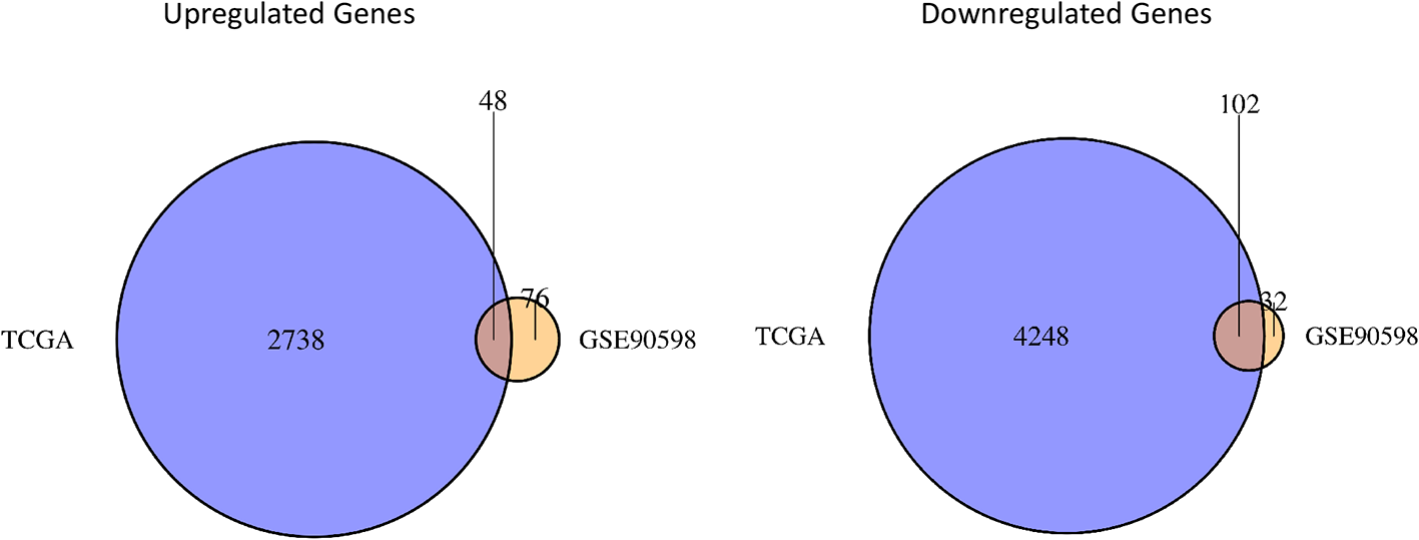
Venn diagrams of differential gene sets. The Venn diagrams show the crossed genes shared by TCGA and GSE90598 glioma datasets. Left panel represents the intersection of two upregulated gene lists, whereas the right panel represents the intersection of two downregulated gene lists. 48 oncogenic genes and 102 suppressor genes are identified in the intersections.

**Figure 2:**
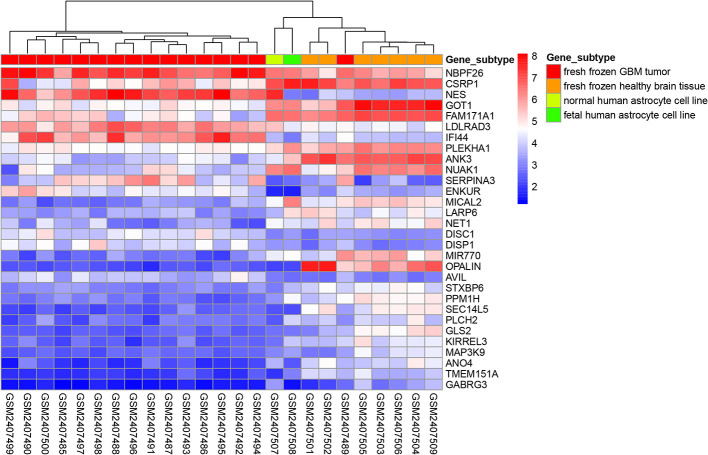
Heatmap of cluster analysis of DEGs expression in the GSE90598 dataset. The heatmap represents the differential expression profiles of DEGs (top 30) in GSE90598 microarray, all the 25 samples are hierarchically clustered. The molecular subtype of each sample was labeled: fresh frozen GBM tissue; fresh frozen healthy brain tissue; normal human astrocyte cell line; fetal human astrocyte cell line.

The “limma” package in R was used to perform differential expression analysis on the GSE90598 dataset (**Supplemental Table 2**), selecting Benjamini’s corrected P value <1e-^10-2^ and fold change of more than 2 as the thresholds. The screening results showed that the 258 DEGs included 124 upregulated genes and 134 downregulated genes. Then, we used the “DESeq2” package in R to analyze the DEGs between the TCGA-GBM and TCGA-LGG datasets (**Supplemental Table 3**), selecting Benjamini’s corrected P value <1e-^10-2^ and fold change of more than 2 as the threshold, and obtained 7152 DEGs. Among these DEGs, 2797 genes were upregulated and 4355 genes were downregulated. After intersecting the results for each dataset, 150 DEGs were obtained, of which 48 were upregulated and 102 were downregulated. The results were displayed with the “VennDiagram” package in R using Venn diagrams (**Figure 1**). The results of cluster analysis of the DEGs from the GSE90598 dataset are shown in **Figure 2**. The top 30 DEGs were selected and displayed in the heatmap. Sample types included healthy human brain tissue, GBM tissue and astrocyte cell line samples.

### Gene Ontology and Kyoto Encyclopedia of Genes and Genomes Pathway Analyses of Differentially Expressed Genes

**Figure 3:**
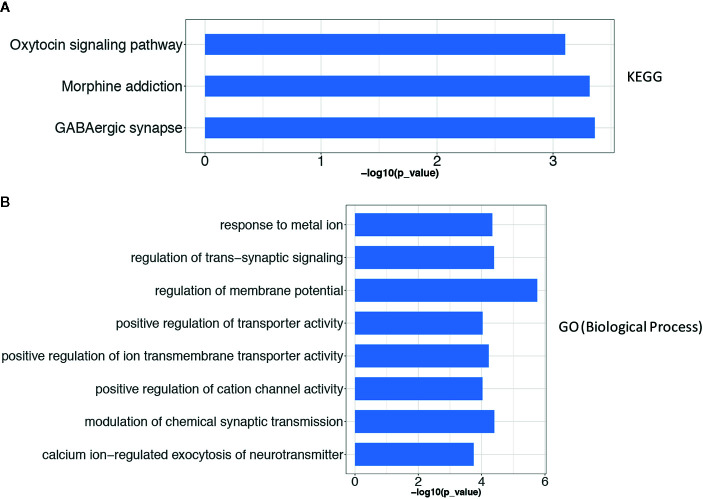
The results of the gene enrichment of identified DEGs. **(A)** KEGG pathway enrichment results; **(B)** GO term (Biological Process) function enrichment results. The Over-Representation method of the “clusterProfiler” package in R was used to enrich the GO and KEGG respectively.

To describe the biological characteristics and functional annotations of the DEGs, the overrepresentation method of the “clusterProfiler” package in R was used to perform GO and KEGG enrichment analyses. The top 10 pathways were sorted by p value and displayed (**Figure 3**). The detailed enrichment results are shown in **Tables 3**, **4**, and **Supplemental Table 4**. In the biological process category of the GO database, the DEGs were mainly enriched in signal pathways such as modulation of chemical synaptic transmission (GO: 0050804), regulation of transsynaptic signaling (GO: 0099177) and positive regulation of transporter activity (GO: 0032411). In the KEGG pathway enrichment results, the DEG-related pathways mainly involved GABAergic synapse (hsa04727), morphine addiction (hsa05032) and the oxytocin signaling pathway (hsa04921).

**Table 3:** GO (Biological Process) function enrichment results (sorted according to p value, the table only shows the top 10 most significant rows) of DEGs from GSE90598 dataset.

ID	Description	p value	p. adjust	Count
GO:0042391	regulation of membrane potential	1.74E-06	0.004	14
GO:0050804	modulation of chemical synaptic transmission	3.96E-05	0.027	12
GO:0099177	regulation of trans-synaptic signaling	4.05E-05	0.027	12
GO:0010038	response to metal ion	4.57E-05	0.027	11
GO:0032414	positive regulation of ion transmembrane transporter activity	5.94E-05	0.028	6
GO:0032411	positive regulation of transporter activity	9.28E-05	0.031	6
GO:2001259	positive regulation of cation channel activity	9.36E-05	0.031	5
GO:0048791	calcium ion-regulated exocytosis of neurotransmitter	1.78E-04	0.052	3
GO:0048167	regulation of synaptic plasticity	2.23E-04	0.058	7
GO:0032412	regulation of ion transmembrane transporter activity	2.82E-04	0.059	8

**Table 4:** KEGG pathway enrichment results of DEGs from GSE90598 dataset.

ID	Description	p value	p. adjust	Count
hsa04727	GABAergic synapse	4.36E-04	0.036	5
hsa05032	Morphine addiction	4.83E-04	0.036	5
hsa04921	Oxytocin signaling pathway	7.85E-04	0.039	6

**Figure 4:**
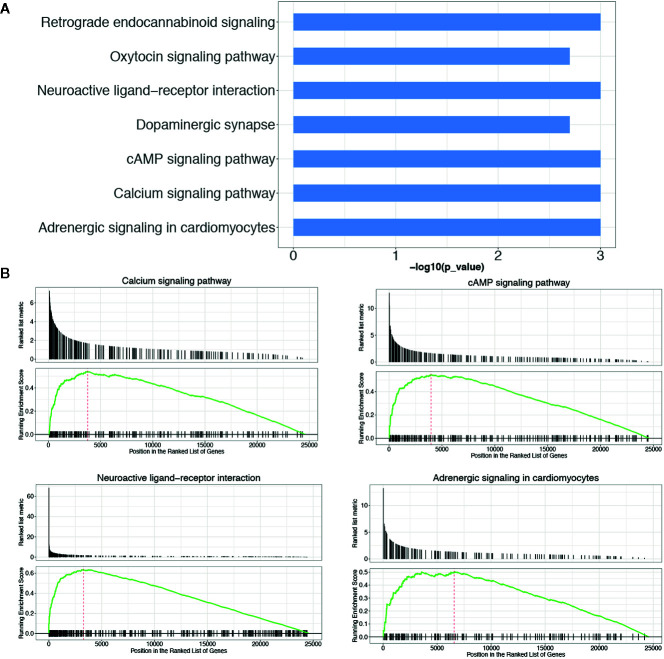
KEGG pathway analysis of DEGs between LGG and GBM subtypes. **(A)** The KEGG pathway enrichment analysis of differential genes between LGG and GBM subtypes; **(B)** GSEA analysis of DEGs significantly expressed between LGG and GBM subtypes using the KEGG pathway datasets.

KEGG pathway enrichment analysis of DEGs between the LGG and GBM subtypes was performed using the GSEA method. The enrichment pathway results are shown in **Figure 4**, **Table 5** and **Supplemental Table 5**. Most of the pathways with different expression levels between LGG and GBM were related to signal transduction.

**Table 5:** The results of KEGG pathway analysis between LGG and GBM by GSEA method.

ID	Description	p value	p. adjust	Enrichment Score
hsa04020	Calcium signaling pathway	9.99E-04	0.020	0.542
hsa04024	cAMP signaling pathway	9.99E-04	0.020	0.549
hsa04080	Neuroactive ligand-receptor interaction	9.99E-04	0.020	0.634
hsa04723	Retrograde endocannabinoid signaling	9.99E-04	0.020	0.554
hsa04261	Adrenergic signaling in cardiomyocytes	3.00E-03	0.035	0.505
hsa04728	Dopaminergic synapse	3.00E-03	0.035	0.524
hsa04921	Oxytocin signaling pathway	3.00E-03	0.035	0.489

### Module Screening From the Protein–Protein Interaction Network

**Figure 5:**
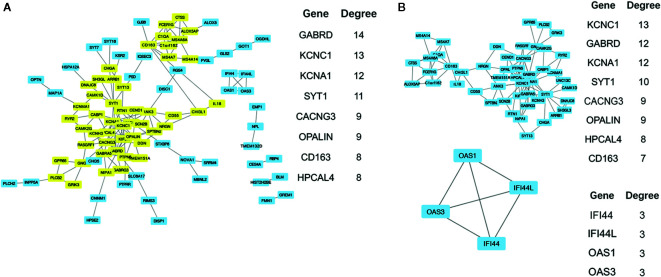
**(A)** The results of PPI network analysis of 150 genes, the major genes (yellow) and their Degree values are listed next. **(B)** Using the MCODE in Cytoscape to analyze the PPI network structure, two main modules are generated. Gene list and their degree values of modules were listed beside.

To obtain the protein interaction network results, further analysis of key gene sets was performed with the protein interaction information in the STRING database. From this analysis, 11 high degree (>= 7) genes were selected as the core gene set, including GABRD, KCNC1, KCNA1, SYT1, CACNG3, OPALIN, CD163, HPCAL4, ANK3, KIF5A, and MS4A6A. The PPI network revealed a total of 153 links. The resulting file was input into Cytoscape for further analysis and mapping (**Figure 5**). Through the MCODE plug-in in Cytoscape, two main modules were obtained. Module 2 contained only four genes, and the links were weak. Therefore, GO term functional enrichment and KEGG pathway enrichment analysis were performed on the gene set in module 1, and the main functional pathways of the module are shown in **Tables 6**, **7**, and **Supplemental Table 6**. In order to further analyze whether these core genes still have significant differences in different histological subtypes of glioma. We performed differential analysis of the transcriptome expression data between LGGs and GBMs from 320 cases of astrocytoma in TCGA datasets, and found that among the 11 core genes selected above, 10 genes: GABRD, KCNA1, SYT1, CACNG3, OPALIN, CD163, HPCAL4, ANK3, KIF5A, and MS4A6A were differentially expressed (**Supplemental Figure 2A**). We also evaluated whether these core genes have significant differences in different molecular subtypes through performing differential analysis of the transcriptome expression data between LGGs and GBMs without IDH mutation or without 1p/19q deletion, and we found that between IDH wild-type TCGA-LGG and TCGA-GBM samples, 8 genes (GABRD, KCNA1, SYT1, CACNG3, OPALIN, HPCAL4, and KIF5A) showed differential expression patterns (**Supplemental Figure 2B**). In addition, there were significant differences in the expression levels of the 4 genes (KCNA1, CACNG3, CD163, and MS4A6A) in the 1p/19q non-co-deleted TCGA-LGG and TCGA-GBM samples (**Supplemental Figure 2C**).

**Table 6:** GO (Biological Process) function enrichment results (sorted according to p value, the table shows only the top 10 most significant rows) of the core gene set in module 1.

ID	Description	p value	p. adjust	Count
GO:0042391	regulation of membrane potential	1.83E-09	2.22E-06	11
GO:0034765	regulation of ion transmembrane transport	5.25E-08	1.78E-05	10
GO:0032412	regulation of ion transmembrane transporter activity	5.42E-08	1.78E-05	8
GO:0022898	regulation of transmembrane transporter activity	6.77E-08	1.78E-05	8
GO:0032414	positive regulation of ion transmembrane transporter activity	7.54E-08	1.78E-05	6
GO:0010038	response to metal ion	8.77E-08	1.78E-05	9
GO:0032409	regulation of transporter activity	1.07E-07	1.85E-05	8
GO:0032411	positive regulation of transporter activity	1.22E-07	1.85E-05	6
GO:2001259	positive regulation of cation channel activity	3.53E-07	4.76E-05	5

**Table 7:** KEGG pathway enrichment results (sorted according to p value, the table shows only the top 10 most significant rows) of the core gene set in module 1.

ID	Description	p value	p. adjust	Count
hsa05032	Morphine addiction	1.01E-05	1.07E-03	5
hsa04723	Retrograde endocannabinoid signaling	1.05E-04	4.01E-03	5
hsa04921	Oxytocin signaling pathway	1.23E-04	4.01E-03	5
hsa04911	Insulin secretion	1.66E-04	4.01E-03	4
hsa04727	GABAergic synapse	1.89E-04	4.01E-03	4
hsa04713	Circadian entrainment	2.64E-04	4.42E-03	4
hsa05033	Nicotine addiction	2.92E-04	4.42E-03	3
hsa04728	Dopaminergic synapse	8.26E-04	1.09E-02	4
hsa04261	Adrenergic signaling in cardiomyocytes	1.34E-03	1.57E-02	4
hsa05152	Tuberculosis	2.62E-03	2.78E-02	4

### Survival Analysis of Putative Hub Genes and Verification

**Figure 6:**
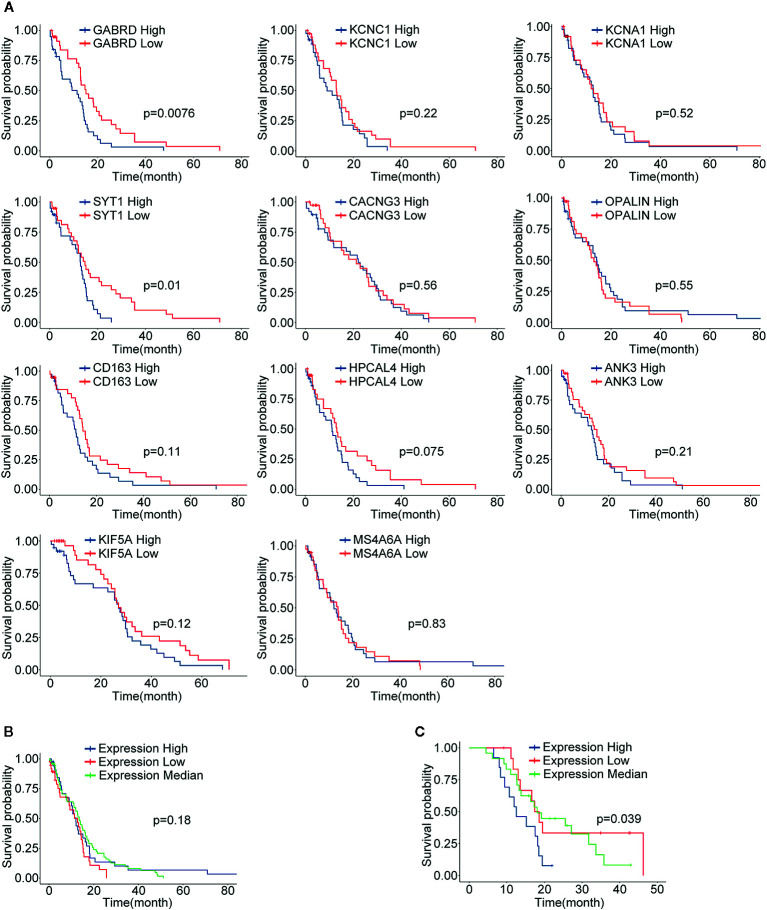
The results of survival analysis of core genes. **(A)** The relationship between the expression level of each gene and the GBM patient’s survival time, blue indicates the high expression group (N=77), red indicates the low expression group (N=77); **(B)** The survival curve and characteristic index of the core genes in the TCGA-GBM group vs. patient survival time; blue indicates the high expression group, red indicates the low expression group, and green indicates the intermediate expression group; **(C)** The results in validation dataset GSE83300, explained as in **(B)**.

Through differential gene analysis and MCODE module analysis, 11 core GBM genes were ultimately determined. According to the expression levels of core genes, population quartiles were used as thresholds, and patients with GBM were divided into core gene high expression and low expression groups. Survival analysis was performed, and the survival curves are shown in **Figure 6A**. Among them, two genes with a significant correlation between gene expression and survival (p value < 0.05) included GABRD and SYT1. However, the relationship between other genes and survival was not very significant. For further analysis, the expression levels of the 11 core genes were combined, and patients were divided into high expression, low expression, and intermediate expression groups according to the overall expression level of the core genes, and survival analysis was performed. The results are shown in **Figure 6B**. When comparing core gene expression and survival, the p value was 0.11, and the correlation was not significant. Subsequently, the survival analysis results were verified in the GSE83300 dataset. By combining the expression levels of core genes, the feature index sj was calculated, and 50 samples were divided into high-, low- and medium-expression groups by quartiles. The p value when comparing core gene expression and survival was 0.039. The expression levels of the 11 screened core genes in the validation group were significantly correlated with patient survival. We analyzed the protein expression level of the 11 core genes in clinical specimens from The Human Protein Atlas. Protein expression level of survival-related gene SYT1 was shown in **Supplemental Figure 3**. It had positive strong expression in GBM cerebral cortex tissues and negative weak expression in LGG cerebral cortex tissues.

### Core Gene Related Drug Targets Screening

The drug related data were downloaded from the DrugBank database and significance analysis was performed by hypergeometric test. The significantly (p value < 0.01) related drugs are shown in **Table 8** and **Supplemental Table 7** and include tetrachlorodecaoxide, ketamine, cinolazepam, quazepam, fludiazepam, clotiazepam, adinazolam, prazepam, estazolam, and halazepam. Among them, tetrachlorodecaoxide is related to the treatment of cancer, and the other drugs are related to the treatment of brain-related diseases such as anxiety and insomnia.

**Table 8:** The results of hypergeometric test for the relationship between core genes and drugs.

drug_name	drug_key	drug_gene_num	core_gene_num	p value
Tetrachlorodecaoxide	DB05389	2	1	1.16E-03
Ketazolam	DB01587	6	1	3.47E-03
Cinolazepam	DB01594	16	1	9.23E-03
Quazepam	DB01589	16	1	9.23E-03
Fludiazepam	DB01567	16	1	9.23E-03
Clotiazepam	DB01559	16	1	9.23E-03
Adinazolam	DB00546	16	1	9.23E-03
Prazepam	DB01588	17	1	9.80E-03
Estazolam	DB01215	17	1	9.80E-03
Halazepam	DB00801	17	1	9.80E-03

## Discussion

A large number of current studies indicate that the occurrence and development mechanism of glioma may be related to the interaction of polygenic genetic factors with environmental factors (14–16). As early as 2002, some researchers developed a classification system based on DNA microarray gene expression data. According to gene expression characteristics, childhood medulloblastoma can be distinguished from other brain tumors, including primitive neuroectodermal tumors (PNETs), atypical teratoid/rhabdoid tumors (AT/RTs) and malignant gliomas (17). Subsequent studies have found that although all belong to the same grade of astrocytoma, patients with different molecular expression patterns have different clinical progression features (18). Furthermore, researchers found that molecular subclasses are closely related to the prognosis, disease progression and treatment of glioma (19–21). With the development of next-generation sequencing technology, neurooncologists have a better understanding of gliomas, especially glioblastomas. IDH1 mutations were found in 12% of glioblastoma patients by second-generation sequencing. Such mutations often occur in young patients and patients with secondary glioblastomas and are associated with favorable overall survival (22). Based on the work of the TCGA network, multidimensional genomic data have been integrated to classify GBM into proneural, neural, classical, and mesenchymal subtypes based on the aberrations and gene expression of EGFR, NF1, and PDGFRA/IDH1. Further research revealed that classical subtype patients benefit the most from aggressive therapy, while proneural subtype patients do no benefit (23). Since then, the clinical importance of tumor protein p53 (TP53) abnormalities, deletions involving chromosomes 1p and 19q, MGMT promoter methylation status, abnormalities in the PTEN tumor suppressor gene and the BRAF oncogene, and IDH mutations in gliomas have become better illuminated (5, 24, 25). This refined classification of glioma into different molecular entities may lead to the use of different treatment regimens (26, 27).

In the present study, we obtained the gene expression profile from the GEO and TCGA databases and then identified the DEGs in glioma using bioinformatics analysis. Functional enrichment analysis showed that these DEGs were mainly involved in signal transduction and similar functional patterns in glioma. Most of these DEGs were mainly related to signal transmission pathways such as modulation of chemical synaptic transmission, regulation of transsynaptic signaling and positive regulation of transporter activity. In addition, the main enriched pathways involving the DEGs were the GABAergic synapse, morphine addiction and oxytocin signaling pathways. Chemical synaptic transmission is one of two main modalities of synaptic transmission. The released neurotransmitter transfers information from one cell to an adjacent cell, which is required for communication between neurons (28). A recent study showed that synaptic transmission plays a role in the pathogenesis of neurological diseases such as epilepsy, and its chemical modulators may become potential therapeutic targets (29). Transsynaptic signals between the pre- and postsynapse are essential for synapse assembly. The dysregulation of transsynaptic signaling could cause severe synapse loss and impair many facets of organization transsynaptically and cell autonomously (30). At the same time, the enriched KEGG pathways, such as the GABAergic synapse and oxytocin signaling pathways, are also mainly related to signal transduction and are involved in neuroanatomy and pathophysiological processes of nervous system diseases (31–33). Reports on signal transduction have mainly focused on neurodevelopment and nervous system diseases such as epilepsy and neurodegenerative diseases, and few reports have mentioned a relation to neurological tumors. However, a recent study showed that antitumor therapy could contribute to seizure control and that antiepileptic drugs might have beneficial effects on tumors, which provides new ideas for future tumor research (34).

The STRING database is an open database used to assess proteins and their functional interactions for a full understanding of biological phenomena (11). In the present study, this PPI database was utilized to construct interactive networks by screening DEGs. Through online analysis, the core gene set containing 11 hub genes, GABRD, KCNC1, KCNA1, SYT1, CACNG3, OPALIN, CD163, HPCAL4, ANK3, KIF5A, and MS4A6A, was identified. Among the core genes, the expression levels of GABRD and SYT1 were closely related to the survival time of glioma patients. GABRD (gamma-aminobutyric acid type A receptor subunit delta) is a member of the γ-aminobutyric acid A (GABAA) receptors, a class of transmembrane ligand-gated chloride channels expressed in the mammalian brain. δGABAA receptors have been reported to participate in certain DG-dependent memory behaviors and facilitate neurogenesis. Gabrd (-/-) mice exhibited impaired recognition memory and contextual discrimination because the migration, maturation, and dendritic development of adult-born neurons were impaired (35). In adult IDH wild-type (WT) diffuse LGG, GABRD expression was independently associated with overall survival (OS) status and showed a moderate negative correlation with tumor-infiltrating macrophages (TIMs) (36). Our analysis showed that among GBM patients, the prognosis of the high GABRD expression group was worse than that of the low expression group. SYT1, synaptotagmin 1, is a calcium-binding protein that triggers fast Ca(2+)-dependent transmitter release in response to membrane depolarization (37). SYT1 not only participates in various biological processes, such as lipid transport (38) and the pathophysiology of neurological diseases (39) but also may play a crucial role in the pathogenesis and treatments of various tumors (40–42). Nord H et al. analyzed the detailed genetic profiling of a set of 78 glioblastomas and found that eight genes, such as SYT1, might be considered novel candidate oncogenes (43). A later study further analyzed more glioblastoma and normal brain tissue samples from the TCGA and GEO databases, and the results showed that SYT1 is a core gene among 552 differentially expressed genes (44). Our findings are similar to those of the two previous studies. KCNC1 encodes KV3.1, a subunit of the KV3 voltage-gated potassium ion channels. A recurrent *de novo* mutation, c.959G>A (p.Arg320His), in KCNC1 was identified as a new major cause for progressive myoclonus epilepsies (PMEs) (45). The voltage-gated K+ channel gene KCNA1 (KV1.1) is also closely related to epilepsy, as confirmed by animal experiments (46). CACNG3, a member of calcium channels, was evaluated as a susceptibility locus by linkage and association analysis in childhood absence epilepsy (CAE) (47). TMEM10/OPALIN, which encodes a novel transmembrane glycoprotein, could be used as a specific marker for myelinating oligodendrocytes and perhaps for the evaluation of myelination diseases (48). Immunohistochemistry experiments revealed that the macrophage scavenger receptor CD163 could contribute to neuropathological changes in “high inflammation” schizophrenia brains (49). The higher expression level of hippocalcin like 4 (HPCAL4), a neural calcium sensor, was detected in the lateral nucleus of the amygdala (LA) with stimuli associated with danger through fear conditioning (50). Ankyrin-3 (ANK3), encoding the adaptor protein Ankyrin-G (AnkG), has been indicated as an important factor in the pathophysiology of schizophrenia and bipolar disorder (51). Kinesin family member 5A (KIF5A) has been reported to regulate neuronal surface expression of GABA(A)Rs *via* an interaction with GABA(A)R-associated protein (GABARAP), indicating that KIF5A could be involved in inhibitory neural transmission regulation related to epilepsy (52). Another hub gene, membrane spanning 4-domain A6A (MS4A6A), has been identified as a new Alzheimer’s disease (AD) susceptibility locus by genome-wide association studies (53). With almost no exceptions, all the above-mentioned hub genes are involved in the process of signal transduction in the nervous system and are related to neurodevelopmental, neurodegenerative and neuropsychiatric disorders. In addition, GABRD and SYT1 are implicated in gliomagenesis, and all other core genes, with the exception of OPALIN, have been reported to play a role in tumor-related diseases, including glioma. As our investigation has shown, abnormal signal transduction is the important property of glioma, and further in-depth understanding of the molecular mechanisms holds promise for the development of cancer drugs that target this process.

Interestingly, the core gene-related drugs were identified by combined significance analysis of the expression of core genes and the data from the DrugBank database. The top 10 candidate drugs filtered by p value included tetrachlorodecaoxide, ketamine, cinolazepam, quazepam, fludiazepam, clotiazepam, adinazolam, prazepam, estazolam, and halazepam. Among them, tetrachlorodecaoxide (TCDO, WF10), a chlorite-based nontoxic compound, is implicated in oncotherapy, and the other drugs are involved in the therapeutic management of brain-related diseases such as anxiety and insomnia. As early as 30 years ago, TCDO was shown to improve the oxygenation status of solid tumors and then sensitize tumors to treatments, such as radiotherapy (54). Animal experiment results showed that TCDO could act as a therapeutic agent in acute radiation syndrome and that it significantly reduced the carcinogenic risk in rats after exposure to ionizing radiation (55). Subsequent researchers designed randomized controlled trials in an attempt to evaluate the effectiveness of TCDO in treating oral mucositis or late hemorrhagic radiation cystitis in patients receiving radiochemotherapy, and the results showed that TCDO significantly reduced the recurrence of objective hematuria but was not found to be effective in treating mucositis (56, 57). Although the other identified drugs have not been reported to play therapeutic roles in human tumors, particularly gliomas, our analysis provided a starting point for understanding the commonalities between nervous system tumors and neurodevelopmental or neurodegenerative diseases. In addition, studies have indicated that antitumor therapy can contribute to seizure control and that antiepileptic drugs might have beneficial effects on tumors (34), which also provides new ideas for the in-depth study of the common molecular mechanisms and shareable therapeutic regimens of both neuropsychiatric diseases and neurological tumors. In summary, our investigation, which is based on a large number of cases and bioinformatics methods, can help researchers reexamine the data from a new perspective. Overall, the prognostic significance and subtype-based expression of hub genes were evaluated in a large cohort and verified experimentally, indicating that these hub genes from the bioinformatics analysis assume on oncogenic role in the progression of glioma.

## Data Availability Statement

The original contributions presented in the study are included in the article/**Supplementary Material**. Further inquiries can be directed to the corresponding author.

## Author Contributions

JY contributed to the publication search, data extraction, draft writing, and conception and design. QY contributed to the quality assessment, conception and design, statistical analysis, and editing. All authors contributed to the article and approved the submitted version.

## Funding

The study was supported by grant from the National Natural Science Foundation of China (81700600 to QY).

## Conflict of Interest

The authors declare that the research was conducted in the absence of any commercial or financial relationships that could be construed as a potential conflict of interest.

## Supplementary Material

Supplementary Figure 1 Flow chart of data preparing, processing and analysis.

Supplementary Figure 2 Volcano plot of DEGs expression in the TCGA-GBM and TCGA-LGG datasets. **(A)** Volcano plot of the DEGs between astrocytomas of TCGA-LGG dataset (n=167) and astrocytomas of TCGA-GBM (n=153) dataset. **(B)** Volcano plot of the DEGs between LGGs without IDH mutation (n=94) and GBMs without IDH mutation (n=140) in the TCGA datasets. **(C)** Volcano plot of the DEGs between LGGs without 1p/19q codeletion (n=343) and GBMs 1p/19q codeletion (n=148) in the TCGA datasets. Red and blue indicate DEGs with a padj < 0.05 and |log2FoldChange| >1. Grey indicates no DEGs or DEGs with a padj ≥ 0.05 and |log2FoldChange| ≤ 1.

Supplementary Figure 3 The expression of SYT1 in cerebral cortex tissue of GBM patient and negative weak expression in cerebral cortex tissue of LGG patient. Images were taken from the Human Protein Atlas (http://www.proteinatlas.org) online database. Four samples of LGG and six samples of GBM were analyzed. The ID numbers of patients with LGG were 122, 164, 2858, and 2890. The ID numbers of patients with GBM were 3, 1587, 1608, 1644, 2811, and 2849. The antibody used in the staining was from Sigma-Aldrich (HPA008394, 1: 90). Antigen retrieval did by HIER pH6.

## References

[1] Ostrom QT Gittleman H Truitt G Boscia A Kruchko C Barnholtz-Sloan JS. CBTRUS Statistical Report: Primary Brain and Other Central Nervous System Tumors Diagnosed in the United States in 2011-2015. Neuro Oncol (2018) 20:v1–v86. 10.1093/neuonc/noy131 PMC6129949 30445539

[2] Sturm D Orr BA Toprak UH Hovestadt V Jones D Capper D. New Brain Tumor Entities Emerge from Molecular Classification of CNS-PNETs. Cell (2016) 164:1060–72. 10.1158/1538-7445.AM2016-2696 PMC5139621 26919435

[3] Molinaro AM Taylor JW Wiencke JK Wrensch MR. Genetic and molecular epidemiology of adult diffuse glioma. Nat Rev Neurol (2019) 15:405–17. 10.1038/s41582-019-0220-2 PMC7286557 31227792

[4] Brat DJ Verhaak RG Aldape KD Yung WK Salama SR Cooper LA. Comprehensive, Integrative Genomic Analysis of Diffuse Lower-Grade Gliomas. N Engl J Med (2015) 372:2481–98. 10.1056/NEJMoa1402121 PMC4530011 26061751

[5] van den Bent MJ Smits M Kros JM Chang SM. Diffuse Infiltrating Oligodendroglioma and Astrocytoma. J Clin Oncol (2017) 35:2394–401. 10.1200/JCO.2017.72.6737 28640702

[6] Wang Y Li S Chen L You G Bao Z Yan W. Glioblastoma with an oligodendroglioma component: distinct clinical behavior, genetic alterations, and outcome. Neuro Oncol (2012) 14:518–25. 10.1093/neuonc/nor232 PMC3309852 22326863

[7] Weller M Stupp R Hegi ME van den Bent M Tonn JC Sanson M. Personalized care in neuro-oncology coming of age: why we need MGMT and 1p/19q testing for malignant glioma patients in clinical practice. Neuro Oncol (2012) 14(Suppl 4):v100–8. 10.1093/neuonc/nos206 PMC3480248 23095825

[8] Ritchie ME Phipson B Wu D Hu Y Law CW Shi W. limma powers differential expression analyses for RNA-sequencing and microarray studies. Nucleic Acids Res (2015) 43:e47. 10.1093/nar/gkv007 25605792 PMC4402510

[9] Love MI Huber W Anders S. Moderated estimation of fold change and dispersion for RNA-seq data with DESeq2. Genome Biol (2014) 15:550. 10.1186/s13059-014-0550-8 25516281 PMC4302049

[10] Yu G Wang LG Han Y He QY. clusterProfiler: an R package for comparing biological themes among gene clusters. OMICS (2012) 16:284–7. 10.1089/omi.2011.0118 PMC3339379 22455463

[11] Szklarczyk D Gable AL Lyon D Junge A Wyder S Huerta-Cepas J. STRING v11: protein-protein association networks with increased coverage, supporting functional discovery in genome-wide experimental datasets. Nucleic Acids Res (2019) 47:D607–13. 10.1093/nar/gky1131 PMC6323986 30476243

[12] Shannon P Markiel A Ozier O Baliga NS Wang JT Ramage D. Cytoscape: a software environment for integrated models of biomolecular interaction networks. Genome Res (2003) 13:2498–504. 10.1101/gr.1239303 PMC403769 14597658

[13] Wishart DS Feunang YD Guo AC Lo EJ Marcu A Grant JR. DrugBank 5.0: a major update to the DrugBank database for 2018. Nucleic Acids Res (2018) 46:D1074–82. 10.1093/nar/gkx1037 PMC5753335 29126136

[14] Ohgaki H Kleihues P. Epidemiology and etiology of gliomas. Acta Neuropathol (2005) 109:93–108. 10.1007/s00401-005-0991-y 15685439

[15] Braganza MZ Kitahara CM Berrington DGA Inskip PD Johnson KJ Rajaraman P. Ionizing radiation and the risk of brain and central nervous system tumors: a systematic review. Neuro Oncol (2012) 14:1316–24. 10.1093/neuonc/nos208 PMC3480263 22952197

[16] Elsherbiny ME Emara M Godbout R. Interaction of brain fatty acid-binding protein with the polyunsaturated fatty acid environment as a potential determinant of poor prognosis in malignant glioma. Prog Lipid Res (2013) 52:562–70. 10.1016/j.plipres.2013.08.004 PMC4461378 23981365

[17] Pomeroy SL Tamayo P Gaasenbeek M Sturla LM Angelo M McLaughlin ME. Prediction of central nervous system embryonal tumour outcome based on gene expression. Nature (2002) 415:436–42. 10.1038/415436a 11807556

[18] Wessels PH Weber WE Raven G Ramaekers FC Hopman AH Twijnstra A. Supratentorial grade II astrocytoma: biological features and clinical course. Lancet Neurol (2003) 2:395–403. 10.1016/S1474-4422(03)00434-4 12849117

[19] Phillips HS Kharbanda S Chen R Forrest WF Soriano RH Wu TD. Molecular subclasses of high-grade glioma predict prognosis, delineate a pattern of disease progression, and resemble stages in neurogenesis. Cancer Cell (2006) 9:157–73. 10.1016/j.ccr.2006.02.019 16530701

[20] Vitucci M Hayes DN Miller CR. Gene expression profiling of gliomas: merging genomic and histopathological classification for personalised therapy. Br J Cancer (2011) 104:545–53. 10.1038/sj.bjc.6606031 PMC3049580 21119666

[21] Mischel PS Cloughesy TF Nelson SF. DNA-microarray analysis of brain cancer: molecular classification for therapy. Nat Rev Neurosci (2004) 5:782–92. 10.1038/nrn1518 15378038

[22] Parsons DW Jones S Zhang X Lin JC Leary RJ Angenendt P. An integrated genomic analysis of human glioblastoma multiforme. SCIENCE (2008) 321:1807–12. 10.1126/science.1164382 PMC2820389 18772396

[23] Verhaak RG Hoadley KA Purdom E Wang V Qi Y Wilkerson MD. Integrated genomic analysis identifies clinically relevant subtypes of glioblastoma characterized by abnormalities in PDGFRA, IDH1, EGFR, and NF1. Cancer Cell (2010) 17:98–110. 10.1016/j.ccr.2009.12.020 20129251 PMC2818769

[24] Bourne TD Schiff D. Update on molecular findings, management and outcome in low-grade gliomas. Nat Rev Neurol (2010) 6:695–701. 10.1038/nrneurol.2010.159 21045797

[25] Melin BS Barnholtz-Sloan JS Wrensch MR Johansen C Il’Yasova D Kinnersley B. Genome-wide association study of glioma subtypes identifies specific differences in genetic susceptibility to glioblastoma and non-glioblastoma tumors. Nat Genet (2017) 49:789–94. 10.1038/ng.3823 PMC5558246 28346443

[26] Van Meir EG Hadjipanayis CG Norden AD Shu HK Wen PY Olson JJ. Exciting new advances in neuro-oncology: the avenue to a cure for malignant glioma. CA Cancer J Clin (2010) 60:166–93. 10.3322/caac.20069 PMC2888474 20445000

[27] Buckner J Giannini C Eckel-Passow J Lachance D Parney I Laack N. Management of diffuse low-grade gliomas in adults - use of molecular diagnostics. Nat Rev Neurol (2017) 13:340–51. 10.1038/nrneurol.2017.54 28497806

[28] Pereda AE. Electrical synapses and their functional interactions with chemical synapses. Nat Rev Neurosci (2014) 15:250–63. 10.1038/nrn3708 PMC4091911 24619342

[29] Yamagata A Fukai S. Insights into the mechanisms of epilepsy from structural biology of LGI1-ADAM22. Cell Mol Life Sci (2020) 77:267–74. 10.1007/s00018-019-03269-0 PMC11104983 31432233

[30] Mosca TJ Hong W Dani VS Favaloro V Luo L. Trans-synaptic Teneurin signalling in neuromuscular synapse organization and target choice. NATURE (2012) 484:237–41. 10.1038/nature10923 PMC3326183 22426000

[31] Mandolesi G Gentile A Musella A Fresegna D De Vito F Bullitta S. Synaptopathy connects inflammation and neurodegeneration in multiple sclerosis. Nat Rev Neurol (2015) 11:711–24. 10.1038/nrneurol.2015.222 26585978

[32] Boldog E Bakken TE Hodge RD Novotny M Aevermann BD Baka J. Transcriptomic and morphophysiological evidence for a specialized human cortical GABAergic cell type. Nat Neurosci (2018) 21:1185–95. 10.1038/s41593-018-0205-2 PMC6130849 30150662

[33] Nardou R Lewis EM Rothhaas R Xu R Yang A Boyden E. Oxytocin-dependent reopening of a social reward learning critical period with MDMA. NATURE (2019) 569:116–20. 10.1038/s41586-019-1075-9 30944474

[34] Huberfeld G Vecht CJ. Seizures and gliomas–towards a single therapeutic approach. Nat Rev Neurol (2016) 12:204–16. 10.1038/nrneurol.2016.26 26965673

[35] Whissell PD Rosenzweig S Lecker I Wang DS Wojtowicz JM Orser BA. gamma-aminobutyric acid type A receptors that contain the delta subunit promote memory and neurogenesis in the dentate gyrus. Ann Neurol (2013) 74:611–21. 10.1002/ana.23941 23686887

[36] Zhang H Zhang L Tang Y Wang C Chen Y Shu J. Systemic screening identifies GABRD, a subunit gene of GABAA receptor as a prognostic marker in adult IDH wild-type diffuse low-grade glioma. BioMed Pharmacother (2019) 118:109215. 10.1016/j.biopha.2019.109215 31545245

[37] Atlas D. The voltage-gated calcium channel functions as the molecular switch of synaptic transmission. Annu Rev Biochem (2013) 82:607–35. 10.1146/annurev-biochem-080411-121438 23331239

[38] Bian X Saheki Y De Camilli P. Ca(2+) releases E-Syt1 autoinhibition to couple ER-plasma membrane tethering with lipid transport. EMBO J (2018) 37:219–34. 10.15252/embj.201797359 PMC5770786 29222176

[39] Baker K Gordon SL Melland H Bumbak F Scott DJ Jiang TJ. SYT1-associated neurodevelopmental disorder: a case series. Brain (2018) 141:2576–91. 10.1093/brain/awy209 PMC6113648 30107533

[40] Li R Chiguru S Li L Kim D Velmurugan R Kim D. Targeting Phosphatidylserine with Calcium-Dependent Protein-Drug Conjugates for the Treatment of Cancer. Mol Cancer Ther (2018) 17:169–82. 10.1158/1535-7163.MCT-17-0092 PMC5752623 28939556

[41] Yang J Hou Z Wang C Wang H Zhang H. Gene expression profiles reveal key genes for early diagnosis and treatment of adamantinomatous craniopharyngioma. Cancer Gene Ther (2018) 25:227–39. 10.1038/s41417-018-0015-4 29681617

[42] Jun HJ Johnson H Bronson RT de Feraudy S White F Charest A. The oncogenic lung cancer fusion kinase CD74-ROS activates a novel invasiveness pathway through E-Syt1 phosphorylation. Cancer Res (2012) 72:3764–74. 10.1158/0008-5472.CAN-11-3990 PMC3753671 22659450

[43] Nord H Hartmann C Andersson R Menzel U Pfeifer S Piotrowski A. Characterization of novel and complex genomic aberrations in glioblastoma using a 32K BAC array. Neuro Oncol (2009) 11:803–18. 10.1215/15228517-2009-013 PMC2802400 19304958

[44] Zhou Y Yang L Zhang X Chen R Chen X Tang W. Identification of Potential Biomarkers in Glioblastoma through Bioinformatic Analysis and Evaluating Their Prognostic Value. BioMed Res Int (2019) 2019:6581576. 10.1155/2019/6581576 31119182 PMC6500689

[45] Muona M Berkovic SF Dibbens LM Oliver KL Maljevic S Bayly MA. A recurrent de novo mutation in KCNC1 causes progressive myoclonus epilepsy. Nat Genet (2015) 47:39–46. 10.1038/ng.3144 25401298 PMC4281260

[46] Glasscock E Qian J Yoo JW Noebels JL. Masking epilepsy by combining two epilepsy genes. Nat Neurosci (2007) 10:1554–8. 10.1038/nn1999 17982453

[47] Everett KV Chioza B Aicardi J Aschauer H Brouwer O Callenbach P. Linkage and association analysis of CACNG3 in childhood absence epilepsy. Eur J Hum Genet (2007) 15:463–72. 10.1038/sj.ejhg.5201783 PMC2556708 17264864

[48] Jiang W Yang W Yang W Zhang J Pang D Gan L. Identification of Tmem10 as a novel late-stage oligodendrocytes marker for detecting hypomyelination. Int J Biol Sci (2013) 10:33–42. 10.7150/ijbs.7526 24391449 PMC3879589

[49] Cai HQ Catts VS Webster MJ Galletly C Liu D O’Donnell M. Increased macrophages and changed brain endothelial cell gene expression in the frontal cortex of people with schizophrenia displaying inflammation. Mol Psychiatry (2020) 25:761–75. 10.1038/s41380-018-0235-x PMC7156343 30214039

[50] Lamprecht R Dracheva S Assoun S LeDoux JE. Fear conditioning induces distinct patterns of gene expression in lateral amygdala. Genes Brain Behav (2009) 8:735–43. 10.1111/j.1601-183X.2009.00515.x PMC3625942 19689454

[51] Hughes T Hansson L Sonderby IE Athanasiu L Zuber V Tesli M. A Loss-of-Function Variant in a Minor Isoform of ANK3 Protects Against Bipolar Disorder and Schizophrenia. Biol Psychiatry (2016) 80:323–30. 10.1016/j.biopsych.2015.09.021 26682468

[52] Nakajima K Yin X Takei Y Seog DH Homma N Hirokawa N. Molecular motor KIF5A is essential for GABA(A) receptor transport, and KIF5A deletion causes epilepsy. Neuron (2012) 76:945–61. 10.1016/j.neuron.2012.10.012 23217743

[53] Hollingworth P Harold D Sims R Gerrish A Lambert JC Carrasquillo MM. Common variants at ABCA7, MS4A6A/MS4A4E, EPHA1, CD33 and CD2AP are associated with Alzheimer’s disease. Nat Genet (2011) 43:429–35. 10.1038/ng.803 PMC3084173 21460840

[54] Mueller-Klieser W Vaupel P. Improvement of tumor spheroid oxygenation by tetrachlorodecaoxide. Int J Radiat Oncol Biol Phys (1987) 13:49–54. 10.1016/0360-3016(87)90259-8 3804815

[55] Kempf SR Port RE Ivankovic S. Anticarcinogenic effect of tetrachlorodecaoxide after total-body gamma irradiation in rats. Radiat Res (1994) 139:226–31. 10.2307/3578668 8052699

[56] Veerasarn V Khorprasert C Lorvidhaya V Sangruchi S Tantivatana T Narkwong L. Reduced recurrence of late hemorrhagic radiation cystitis by WF10 therapy in cervical cancer patients: a multicenter, randomized, two-arm, open-label trial. Radiother Oncol: J Eur Soc Ther Radiol Oncol (2004) 73:179–85. 10.1016/S0167-8140(04)00325-1 15542165

[57] Worthington HV Clarkson JE Eden OB. Interventions for preventing oral mucositis for patients with cancer receiving treatment. Cochrane Database Syst Rev (2007) 17:CD000978. 10.1002/14651858.CD000978.pub3 17943748

