# Supplementary figures and images for: Identification of Core Genes and Screening of Potential Targets in Glioblastoma Multiforme by Integrated Bioinformatic Analysis
Source: Front Oncol. 2021 Feb 24;10:615976. doi: 10.3389/fonc.2020.615976 (PMC7943725; doi:10.3389/fonc.2020.615976)

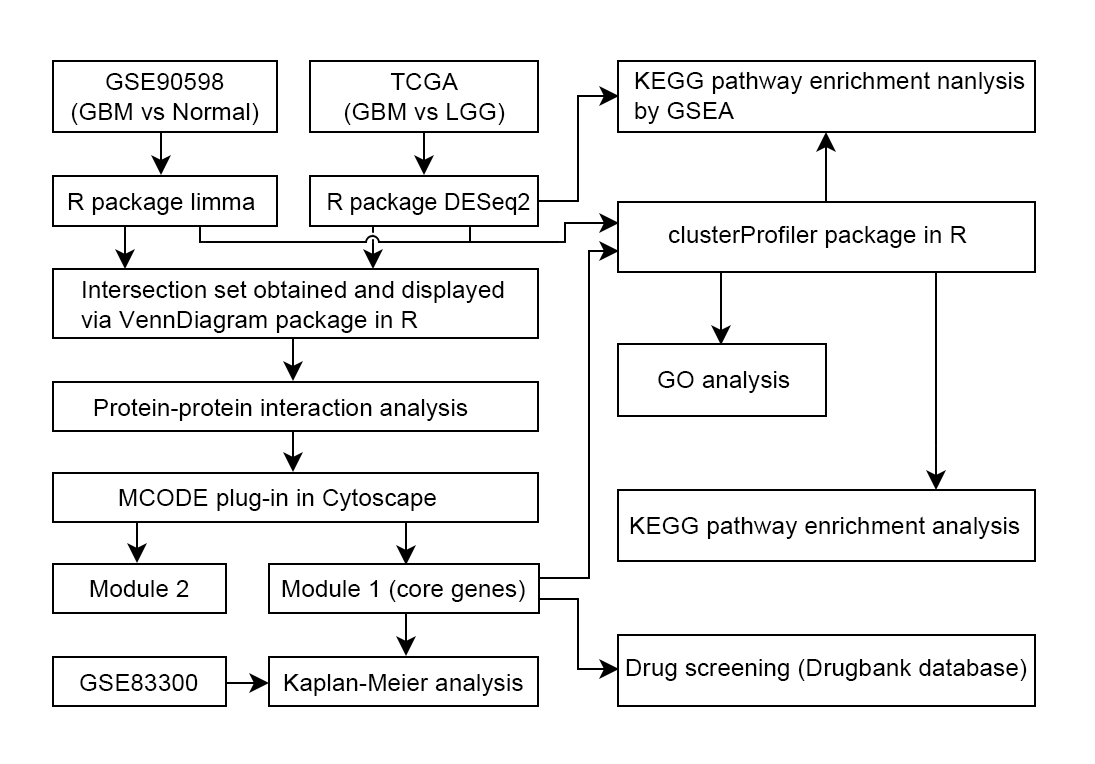

Supplement: Supplementary Figure 1 — Flow chart of data preparing, processing and analysis. [file Image_1.tif]

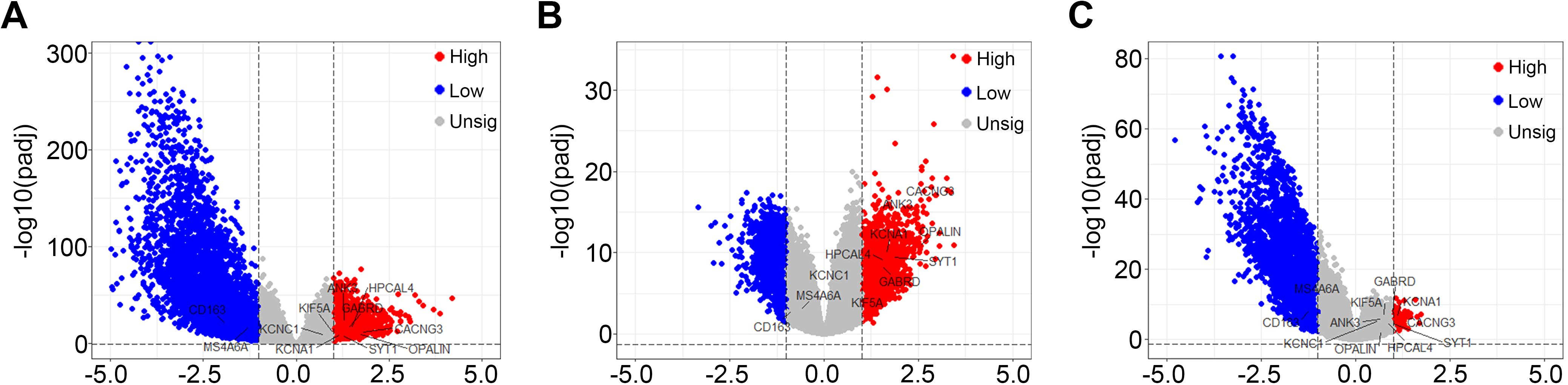

Supplement: Supplementary Figure 2 — Volcano plot of DEGs expression in the TCGA-GBM and TCGA-LGG datasets. (A) Volcano plot of the DEGs between astrocytomas of TCGA-LGG dataset (n=167) and astrocytomas of TCGA-GBM (n=153) dataset. (B) Volcano plot of the DEGs between LGGs without IDH mutation (n=94) and GBMs without IDH mutation (n=140) in the TCGA datasets. (C) Volcano plot of the DEGs between LGGs without 1p/19q codeletion (n=343) and GBMs 1p/19q codeletion (n=148) in the TCGA datasets. Red and blue indicate DEGs with a padj < 0.05 and |log2FoldChange| >1. Grey indicates no DEGs or DEGs with a padj ≥ 0.05 and |log2FoldChange| ≤ 1. [file Image_2.tif]

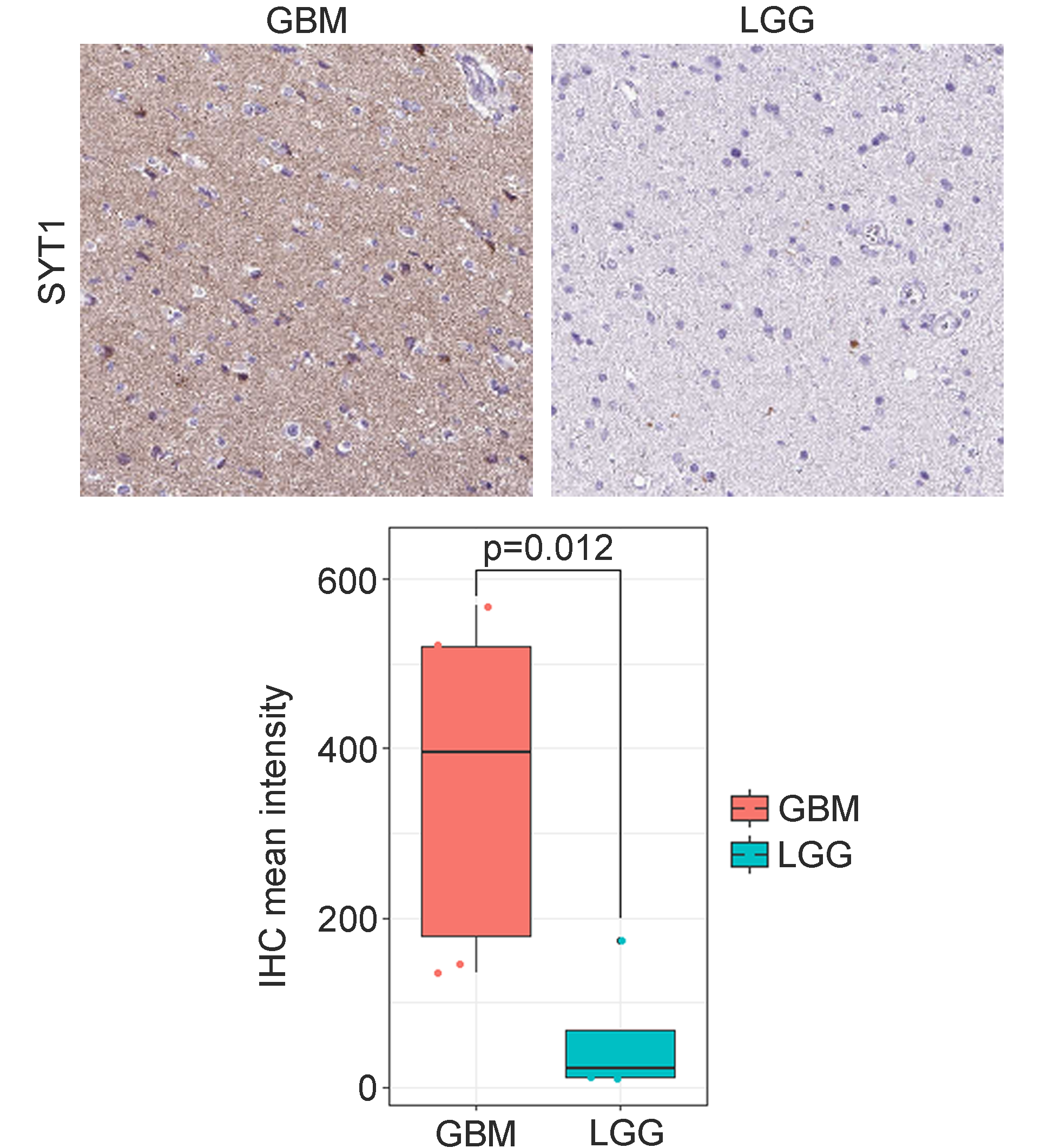

Supplement: Supplementary Figure 3 — The expression of SYT1 in cerebral cortex tissue of GBM patient and negative weak expression in cerebral cortex tissue of LGG patient. Images were taken from the Human Protein Atlas (http://www.proteinatlas.org) online database. Four samples of LGG and six samples of GBM were analyzed. The ID numbers of patients with LGG were 122, 164, 2858, and 2890. The ID numbers of patients with GBM were 3, 1587, 1608, 1644, 2811, and 2849. The antibody used in the staining was from Sigma-Aldrich (HPA008394, 1: 90). Antigen retrieval did by HIER pH6. [file Image_3.tif]
