# Supplementary material for: Identification of Core Genes and Screening of Potential Targets in Glioblastoma Multiforme by Integrated Bioinformatic Analysis
Source: Front Oncol. 2021 Feb 24;10:615976. doi: 10.3389/fonc.2020.615976 (PMC7943725; doi:10.3389/fonc.2020.615976)
Supplement: Supplementary file 4 [file Table_1.docx]

Supplemental Table 1. Baseline characteristics of participants from TCGA datasets

|  | Lower Grade Glioma | Glioblastoma multiforme | Total | P Value |
| --- | --- | --- | --- | --- |
| **Gender** |  |  |  |  |
|  | LGG (n=453) | GBM (n=153) | Total (n=606) |  |
| female | 199 (43.9%) | 54 (35.3%) | 253 (41.7%) |  |
| male | 254 (56.1%) | 99 (64.7%) | 353 (58.3%) | 0.08 |
| **Age** |  |  |  |  |
|  | LGG | GBM |  |  |
| Mean (SD) | 43.2 (13.5) | 59.7 (13.6) |  |  |
| Median [MIN,MAX] | 41 [14,87] | 60 [21,89] |  |  |
| **Age(year)** * |  |  |  |  |
|  | LGG (n=453) | GBM (n=153) | Total (n=606) |  |
| <60 | 64 (14.1%) | 82 (53.6%) | 146 (24.1%) |  |
| >=60 | 389 (85.9%) | 71 (46.4%) | 460 (75.9%) | 1.66e-22 |
| **Histology** |  |  |  |  |
|  | LGG(n=453) | GBM(n=153) | Total (n=606) |  |
| astrocytoma | 167 (36.9%) | 153 (100.0%) |  |  |
| oligoastrocytoma | 112 (24.7%) |  |  |  |
| oligodendroglioma | 174 (38.4%) |  |  |  |
| **IDH status*** |  |  |  |  |
|  | LGG (n=508) | GBM (n=150) | Total (n=658) |  |
| Mutant | 414 (81.5%) | 10 (6.7%) | 424 (64.4%) |  |
| WT | 94 (18.5%) | 140 (93.3%) | 234 (35.6%) | 8.66e-63 |
| **1p/19q codeletion** |  |  |  |  |
|  | LGG(n=511) | GBM(n=148) | Total(n=659) |  |
| codel | 168 (33.1%) |  |  |  |
| non-codel | 343 (67.5%) | 148 (98.7%) | 491 (74.6%) |  |

Note: *: significant
